# The Costliest Gift

**Published:** 1891-04

**Authors:** Louise Chandler Moulton


### THE COSTLIEST GIFT.

I give you a day of my life—
  Treasure no gold could buy—
For peasant and peer are at one
  When the time comes to die;
And all that the monarch has,
  His koh-i-noor or his crown,
He would give for one more day
  Ere he lay his sweet life down.

They are winged, like the viewless wind—
  These days that come and go—
And we count them, and think of the end,
  But the end we cannot know:
The whole world darkens with pain
  When a sunset fades in the west—
.... I give you a day of my life,
  My uttermost gift and my best.

LOUISE CHANDLER MOULTON, *in Youths' Companion.*

